# Comparison of saliva with healthcare workers- and patient-collected swabs in the diagnosis of COVID-19 in a large cohort

**DOI:** 10.1186/s12879-021-06343-w

**Published:** 2021-07-05

**Authors:** Mitnala Sasikala, Yelamanchili Sadhana, Ketavarapu Vijayasarathy, Anand Gupta, Sarala Kumari Daram, Naveen Chander Reddy Podduturi, Duvvur Nageshwar Reddy

**Affiliations:** 1 Department of Molecular Biology, Institute of Translational Research, Asian Healthcare Foundation, AIG Hospitals, Survey No 136, Plot No 2/3/4/5, 1, Mindspace Road, Gachibowli, Hyderabad, Telangana 500032 India; 2 Department of Microbiology, AIG Hospitals, Survey No 136, Plot No 2/3/4/5, 1, Mindspace Road, Gachibowli, Hyderabad, Telangana 500032 India; 3 Department of Critical Care Medicine, AIG Hospitals, Survey No 136, Plot No 2/3/4/5, 1, Mindspace Road, Gachibowli, Hyderabad, Telangana 500032 India; 4 Department of Medical Gastroenterology, AIG Hospitals, Survey No 136, Plot No 2/3/4/5, 1, Mindspace Road, Gachibowli, Hyderabad, Telangana 500032 India

**Keywords:** SARS-COV-2, Saliva, Healthcare worker, Nasopharyngeal swab

## Abstract

**Background:**

A considerable amount of evidence demonstrates the potential of saliva in the diagnosis of COVID-19. Our aim was to determine the sensitivity of saliva versus swabs collected by healthcare workers (HCWs) and patients themselves to assess whether saliva detection can be offered as a cost-effective, risk-free method of SARS-CoV-2 detection.

**Methods:**

This study was conducted in a hospital involving outpatients and hospitalized patients. A total of 3018 outpatients were tested. Of these, 200 qRT-PCR-confirmed SARS-CoV-2-positive patients were recruited for further study. In addition, 101 SARS-CoV-2-positive hospitalized patients with symptoms were also enrolled in the study. From outpatients, HCWs collected nasopharyngeal swabs (NPS), saliva were obtained. From inpatients, HCWs collected swabs, patient-collected swabs, and saliva were obtained. qRT-PCR was performed to detect SARS-CoV-2 by TAQPATH assay to determine the sensitivity of saliva detection. Sensitivity, specificity and positive/negative predictive values (PPV, NPV) of detecting SARS-CoV-2 were calculated using MedCalc.

**Results:**

Of 3018 outpatients (asymptomatic: 2683, symptomatic: 335) tested by qRT-PCR, 200 were positive (males: 140, females: 60; aged 37.9 ± 12.8 years; (81 asymptomatic, 119 symptomatic). Of these, saliva was positive in 128 (64%); 39 of 81 asymptomatic (47%),89 of 119 symptomatic patients (74.8%). Sensitivity of detection was 60.9% (55.4–66.3%, CI 95%), with a negative predictive value of 36%(32.9–39.2%, CI 95%).Among 101 hospitalized patients (males:65, females: 36; aged 53.48 ± 15.6 years), with HCW collected NPS as comparator, sensitivity of saliva was 56.1% (47.5–64.5, CI 95%), specificity 63.5%(50.4–75.3, CI95%) with PPV of 77.2% and NPV of 39.6% and that of self-swab was 52.3%(44–60.5%, CI95%), specificity 56.6% (42.3–70.2%, CI95%) with PPV 77.2% and NPV29.7%. Comparison of positivity with the onset of symptoms revealed highest detection in saliva on day 3 after onset of symptoms. Additionally, only saliva was positive in 13 (12.8%) hospitalized patients.

**Conclusion:**

Saliva which is easier to collect than nasopharyngeal swab is a viable alternate to detect SARS-COV-2 in symptomatic patients in the early stage of onset of symptoms. Although saliva is currently not recommended for screening asymptomatic patients, optimization of collection and uniform timing of sampling might improve the sensitivity enabling its use as a screening tool at community level.

## Background

Accurate detection of SARS-CoV-2 infection is essential for containing the ongoing COVID-19 pandemic. Currently reverse transcriptase-polymerase chain reaction (RT-PCR) performed on swabs obtained from nasopharynx and/or oropharynx are the first line of samples in the diagnosis of COVID-19, although many reports indicate the use of saliva. Collection of saliva is more easy and pleasant for patient than nasopharyngeal swab collection in the diagnosis of COVID-19. It reduces the burden on the healthcare system, negates the need for direct interaction of healthcare workers with patients and decreases the costs involved [1–8]. Swabs collected by patients themselves are demonstrated to lessen the exposure of healthcare workers and the risk of becoming infected [9]. These findings emphasize the need for evaluating the sensitivity of detecting SARS-CoV-2 infection in saliva and patient-collected swabs involving large cohorts of patients, as the reported sensitivities vary [10–18]. Moreover, the sensitivity of detecting SARS-CoV-2 using saliva samples in asymptomatic and symptomatic COVID-19 patients needs to be determined before implementing saliva as a screening tool in clinical practice or to screen population at large. Therefore, our aim was to test the reliability of saliva and swabs collected by healthcare workers and patients themselves in the detection of SARS-CoV-2.

### Patients

This was a prospective, single-center, observational study conducted at AIG Hospitals, Hyderabad, in accordance with the Declaration of Helsinki, approved by the Institutional Ethics Committee. We separately studied inpatients and out patients. A total of 101 qRT-PCR-confirmed COVID-19 patients with clinical symptoms, such as fever, sore throat, cough, and breathlessness, admitted between August 20,2020 to September 20,2020 were enrolled in the study. Patients with clinical symptoms of flu, negative by qRT-PCR and with normal CT scan studies were excluded. We also screened outpatients attending the COVID-19 testing center (*n* = 3018) with and without clinical symptoms. All the patients provided informed consent. Patient demographics, presence or absence of clinical symptoms, duration onset of symptoms, and travel history were recorded. Clinical features of patients are shown in Table 1.

**Table 1:** Clinical features of participants in the study

	OP (***n*** = 200)	IP (***n*** = 101)	Significance
*Age (years)*	37.9 + 12.8	53.48 + 15.6	0.0001
**Gender**			
*Male*	140 (70%)	65 (64.4%)	0.32
*Female*	60 (30%)	36 (35.6%)	0.32
*Asymptomatic*	81 (40.5%)	0	0.0001
*Symptomatic*	119 (59.5%)	101 (100%)	0.0001
**Disease Severity**			
*Mild Symptoms*	119 (59.5%)	90 (89%)	0.0001
*Moderate Disease*	nil	8 (7.9%)	0.0001
*Severe Disease*	nil	3 (2.9%)	0.01
**Comorbidities**			
*Diabetes*	5 (2.5%)	10 (9.9%)	0.005
*Hypertension*	5 (2.5%)	14 (13.9%)	0.0001
*DM + HTN*	6 (3%)	23 (22.8%)	0.0001

*OP* out patients, *IP* in patients (hospitalized patients)

Student t test and Z proportion test used to calculate significance

## Methods

### Swab and saliva collection

Nasopharyngeal swabs were collected by nursing staff from hospitalized patients and by healthcare workers from outpatients. Hospitalized patients were given instructions by the clinical teams for collecting oropharyngeal swabs by themselves. The specimen required were collected on the same day after COVID-19 was confirmed at hospital admission by qRT-PCR. All the patients were asked to collect 2–3 ml of saliva by coughing in sterile urine containers before naso/oropharyngeal swabs were collected. Swabs were placed in sterile viral transport medium, sealed, sent to the inhouse virology laboratory for qRT-PCR. Saliva was not put in viral transport medium, as it has been demonstrated that SARS-CoV-2 RNA is stable in saliva [1]. qRT-PCR was conducted for saliva samples of patients whose NPS were positive for SARS-CoV-2 within 24 h.

### SARS-CoV-2 detection

Total nucleic acid was extracted from 200 μl of viral transport media from the naso/oropharyngeal swabs or 200 μl of whole saliva using a MagMAX Viral Nucleic.

Acid Isolation Kit (Thermo Fisher Scientific) on BIOMEK I series liquid handlers (Beckman, USA) following the manufacturer’s instructions. Nuclei acid was eluted in 50 μl of elution buffer. For SARS-CoV-2 RNA detection, 7 μl of eluted RNA template was used for qRT-PCR using the commercially available TAQPATH kit (Thermo Fisher Scientific, USA) employing QUANTSTUDIO 6.0. TAQPATH uses 3 gene primer/probe sets for the Nucleocapsid gene (N), Spike protein gene (s) and Open Reading Frame gene (ORF), as well as an internal control. Samples were reported positive when all 3 genes were amplified [19].

### Statistics

All patient data were entered into MS-Excel for analysis. Sensitivity, specificity, positive predictive value (PPV), and negative predictive value (NPV) of saliva were assessed against NPS as a reference standard among outpatients and inpatients using MedCalc software.

## Results

Out of 3018 outpatients screened for SARS-CoV-2 by qRT-PCR, 200 patients were positive by nasopharyngeal swab testing (males: 140, females: 60; aged 37.9 ± 12.8 years). Among 200 outpatients, 81 were asymptomatic but tested themselves because of travel or contact history, and 119 tested themselves due to mild symptoms such as dry cough, fever, headache and myalgia. All 101 patients admitted to the hospital (males: 65, females: 36; aged 53.4 ± 15.6 years) were qRT-PCR confirmed for COVID-19 and had either moderate or severe symptoms (Table 1).

**Fig. 1:**
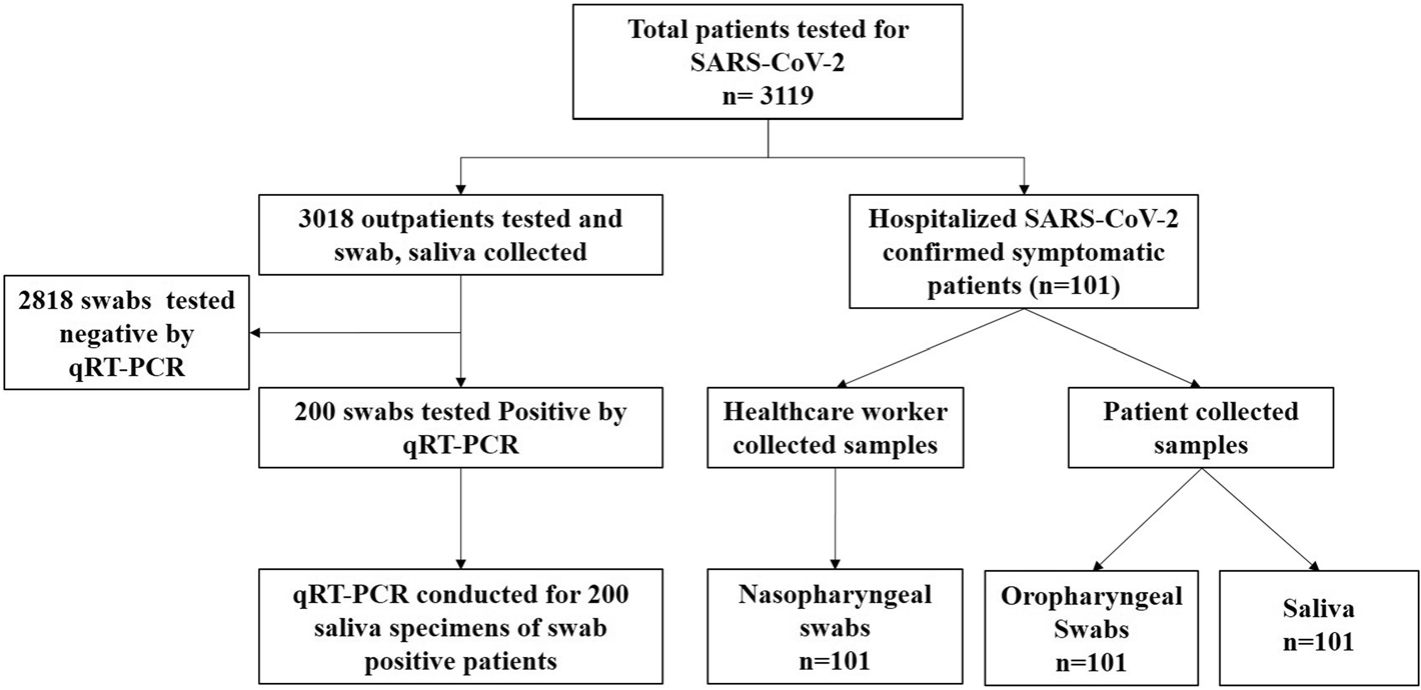
Flow of participants in the study

A total of 301 COVID-19 patient swabs and saliva samples (301 swabs collected by HCWs 101 self-swabs collected by patients, and 301 saliva samples) were analyzed by qRT-PCR (Fig. 1).

**Fig. 2:**
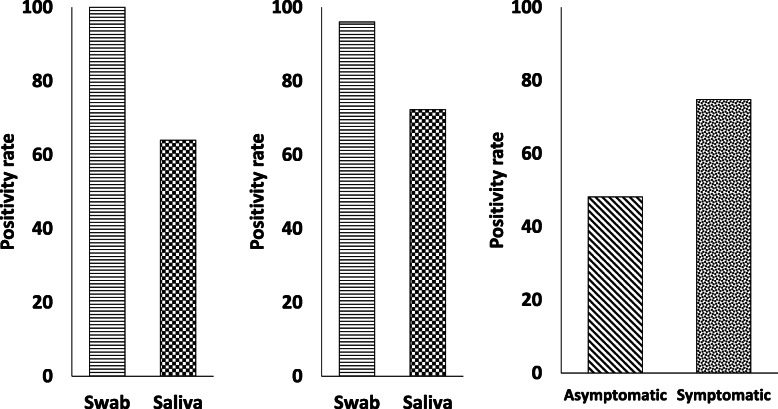
Comparison of Nasopharyngeal swab with saliva. Nasopharyngeal swabs and saliva were tested in outpatients: Left panel shows overall positivity of swabs to be higher in comparison to saliva. Middle panel shows comparison of saliva in swab positive patients with higher detection in swabs.Right panel shows higher positivity in symptomatic patients in comparison to asymptomatic patients

From the 301 COVID-19 patients tested, 278 (92.4%) NPS and 189 (62.8%) saliva samples were positive by qRT-PCR. Among the 200 outpatients whose nasopharyngeal swabs were positive, only 128 (64%) saliva samples tested positive (Fig. 2). Of these, 39 saliva samples were from asymptomatic patients (48.1%:39/81), and 89 were from symptomatic patients (74.78%:89/119). Sensitivity of detection in outpatients was 60.9% (95% CI: 55.4–66.3%) and specificity was 100% ((95% CI: 95–100%) with a NPV of 36%(95% CI: 32.9–39.2%) in saliva samples. Among 101 hospitalized patients with moderate-to-severe disease, swabs were positive in 78 patients (77.2%), and saliva samples were positive in 61 patients (60.4%) and patient collected swabs were positive in 71%. Sensitivity of detection in saliva was 56.1% (95% CI: 47.5–64.5%) and specificity was 63.5%(95% CI: 50.4–75.3%), yielding a PPV of 77.2%(95% CI: 70.4–82.9) and NPV of 39.6%(95% CI: 33.5–46%) and sensitivity of detection. in self-swab was 52.3% (95% CI: 44–60.5%) and specificity was 56.6%(95% CI: 42.3–70.2%), yielding a PPV of 77.2%(95% CI: 70.6–82.7) and NPV of 29.7%(95% CI: 24–36.1%). Additionally, 13 patient saliva samples were positive for SARS-CoV-2, whose nasopharyngeal swabs were negative in symptomatic hospitalized patients.

Analysis of matched samples revealed that 178 out of 301 (59.6%) patients tested positive both by swabs and saliva. Among these 178 patients, 42 were asymptomatic, and 136 were symptomatic. Forty-two out of 81 (51.8%) asymptomatic and 136 out of 217 (62.3%) symptomatic patient samples were qRT-PCR positive by both swab and saliva. Analysis was also conducted for the self-swabs collected by patients themselves along with swabs collected by HCWs and saliva samples in the hospitalized patients. All three samples (HCW-collected swabs, self-swabs and saliva) were positive in 44.5% of patients enrolled. HCW-collected swabs were positive in 78 patients (77.2%), self-swabs were positive in 71 patients (70.3%), and saliva was positive in 61 patients (60.4%).

**Fig. 3:**
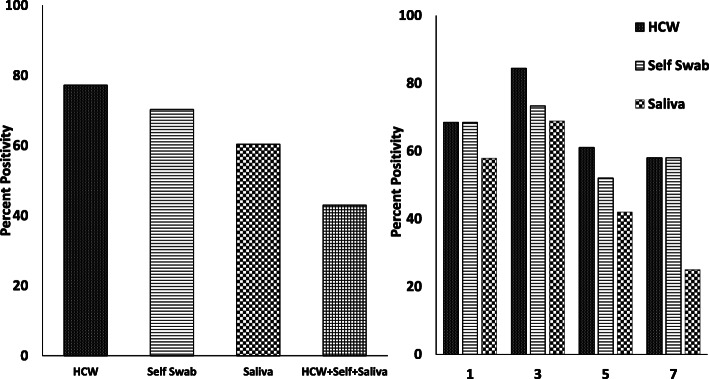
Comparison of nasopharyngeal swabs collected by healthcare workers (HCWs) and self-swabs vs saliva. The left panel shows positivity in all three specimens, and the right panel shows higher detection rates in saliva between 3 and 5 days of onset of symptoms

Comparison of positivity with the onset of symptoms from day 1 to day 7 shows that the positive detection rate of 68.4% was similar in swabs collected by healthcare workers and self-swabs by the patient, while saliva was positive in 57.8% on day 1. The detection rate was highest on day 3 after the onset of symptoms: healthcare worker (HCW)-collected swabs in 84.4%, self-swabs in 73.3% and saliva in 68.8% were positive. On day 7, the positivity was 58% in both swabs collected by healthcare workers and self-swabs, similar to day 1, whereas saliva was positive in 25% of patients (Fig. 3).

**Fig. 4:**
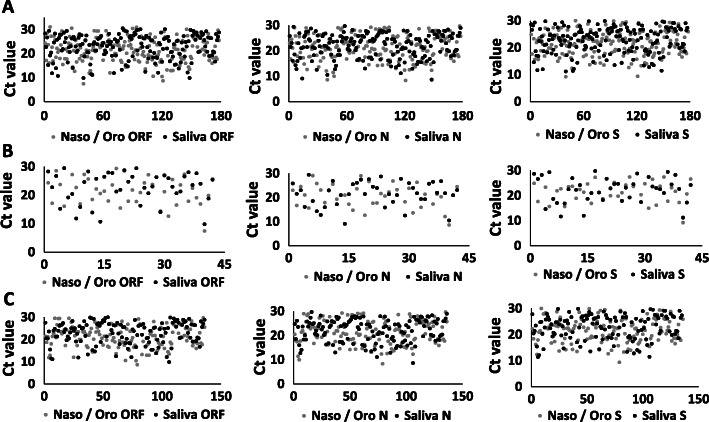
Matched patients swab and saliva viral genes. Scatter plots of Ct values of ORF, N and S genes in patients whose swab and saliva were positive. Panel **A**; Ct values of the three genes in all the patients, Panel **B**; Ct values of three genes in asymptomatic patients, Panel **C**; Ct values of three genes in symptomatic patients. X axis represents Ct values of N, ORF, S genes and Y axis represents the number of patients. Ct values of the 3 genes did not show any significant difference in asymptomatic patients, while in symptomatic patients, Ct values of the ORF (*p* = 0.002) and S (*p* = 0.0007) genes were significantly lower in swabs than saliva samples, while the N gene had no significant difference

All 3 genes (ORF, N and S genes) were amplified in swabs collected by HCWs, self-swabs and saliva samples (Fig. 4). The ORF and S genes showed significantly lower Ct values in swabs than in saliva samples: ORF; *p* = 0.002, S; *p* = 0.004. The N gene did not show significant differences in Ct values. Ct values of the 3 genes did not show any significant difference in asymptomatic patients, while in symptomatic patients, Ct values of the ORF (*p* = 0.002) and S (*p* = 0.0007) genes were significantly lower in swabs than saliva samples, while the N gene had no significant difference.

## Discussion

In this study, overall, we detected SARS-CoV-2 in 64% of saliva specimens from outpatients and 60.4% in saliva specimens from hospitalized patients, while the detection was 77% in NPS collected by HCW. This result indicates that saliva is less sensitive when compared to NPS and corroborates with a report demonstrating 17% greater sensitivity in NPS of hospitalized patients [20]. We also observed that the highest number of patients detected to be SARS-CoV-2 positive in saliva (68%) was on day 3 after the onset of symptoms. This finding suggests the probability of detecting SARS-CoV-2 viral RNA to be greater during early phase of onset of symptoms in hospitalized patients. These results are in agreement with an earlier report indicating the use of saliva to detect SARS-CoV-2 in symptomatic patients [20, 21]. Our study involved outpatients, including asymptomatic individuals, and hospitalized patients with mild-to-moderate symptoms and demonstrated only 51.8% detection in asymptomatic patients. These results are in accordance with the results of Becker et al., who recruited a small number of patients from the community and demonstrated saliva to be less sensitive in asymptomatic patients than in symptomatic patients [22]. We tested a larger number of patients and confirmed that saliva is less sensitive in asymptomatic patients. A sensitivity of 100% detection in saliva samples was reported in hospitalized patients with severe and very severe disease [3]. Our study did not include severe/very severe disease patients needing Intensive Care Unit admission. Another study on a preprint server shows saliva to be more sensitive than nasopharyngeal swabs in symptomatic patients [23]. Our study included both asymptomatic and symptomatic patients and the results obtained indicate that saliva can be used only during early phase of onset of symptoms. It is also known that none of the methods or the samples test 100% positive in detecting SARS-CoV-2 and our results are consistent with these previous reports [24]. A detection rate of 51.8% in asymptomatic patients does not indicate that saliva can be used to screen communities or populations at large for detection of SARS-CoV-2. Nonetheless, saliva can be used as an alternate specimen for SARS-CoV-2 testing, especially in elderly individuals with symptoms who will not be able to move as it is easy to collect, less risky and economical.

Comparison of swabs collected by HCWs with patient-collected self-swabs and saliva also demonstrated that the detection of SARS-CoV-2 in saliva was lower in comparison to NPS, self-swab being the lowest. The significantly lower Ct values of ORF and S genes in swabs demonstrates higher viral loads in NPS than in saliva samples. The higher sensitivity of SARS-CoV-2 detection in saliva samples of outpatients (60.9, 95% CI: 55.4–66.3%) in comparison to hospitalized patients (56.1% (95% CI: 47.5–64.5%) establishes that sensitivity of detecting.

SARS-CoV-2 in saliva is higher in early phase of infection. However, 12.8% positivity only in saliva samples of hospitalized patients in this study is similar to a report from Canada [20] and suggest that saliva testing is to be considered in symptomatic patients when the NPS does not detect SARS-CoV-2. Also, addition of testing saliva along with swabs would increase the detection rate and decrease false negativity. As demonstrated in this study, positivity up to 12.8% in the saliva of patients who tested negative in naso/oropharyngeal swabs would have an additive effect on the detection of SARS-CoV-2 (77.2 + 12.8 = 90%) if saliva can be added to viral transport medium in the same collection tube.

A limitation is that it is not a population/screening study, although we have screened a large cohort. We included all patients who were hospitalized with moderate symptoms and those who attended the outpatient COVID-19 testing center with (mild symptoms) and without symptoms (asymptomatic).

Todate, the detection rate of SARS-CoV-2 in saliva is reported to have a wide range of sensitivity (60–100%). In general, differences in the positivity rate could be due to inherent problems associated with inter individual variations in the technique of swabbing (site/time) across studies, saliva collection (simple spitting/deep coughing), time of saliva collection (morning saliva was demonstrated to have higher viral loads), the type of preservative used (or not used), differences in storage of specimens, etc. Additionally, severity of disease differs among different populations. All the patients enrolled in this study presented with mild to moderate symptoms and it is reported that the disease is predominantly milder in India. All the samples were collected by trained technical staff and the amplification of all three genes in the samples that tested positive eliminates the possibility of technical errors. Mild disease and random saliva sampling without any restrictions on water intake could have led to lower sensitivity in our study. Therefore, practicing uniform protocol worldwide would decrease the conflicting sensitivities and may increase the chances of utilizing this simple, economical, less risky technique in detecting SARS-CoV-2 viral RNA. Such an improved protocol can then be used to test high risk communities, people with flu like symptoms and hospitalized patients who test negative by RT-PCR by NPS.Instead of repeated testing, addition of saliva into VTM tubes with NPS improves sensitivity of detection.

## Conclusion

Our study demonstrates that saliva, easier to collect than Nasopharyngeal swab and Oropharyngeal swab can be used as a specimen in testing symptomatic patients in the early stages of onset of symptoms. Currently, saliva may not be recommended as a testing tool in asymptomatic patients or for screening purposes due to non-availability of a uniform protocol. Our results also demonstrate that testing saliva in swab-negative symptomatic patients would improve detection rates and decrease false negative results.

## Data Availability

The data analysed during the current study are available from the corresponding author on reasonable request.

## Abbreviations

SARS-CoV-2: Severe acute respiratory syndrome corona virus-2
HCW: Healthcare workers
NPS: Nasopharyngeal swab
qRT-PCR: Quantitative reverse transcriptase polymerase chain reaction
PPV: Positive predictive value
NPV: Negative predictive value
N: Nucleocapsid gene
S: Spike gene
ORF: Open Reading Frame gene
